# Effects of physical exercise on college students’ sense of meaning in life: the chain mediating role of stress perception and mental toughness

**DOI:** 10.3389/fpsyg.2025.1612957

**Published:** 2025-06-04

**Authors:** Yong Jiang, Yue Cao

**Affiliations:** School of Physical Education, Liaoning Normal University, Dalian, China

**Keywords:** physical exercise, sense of meaning in life, stress perception, mental toughness, intermediary effect

## Abstract

Physical exercise has a significant effect on college students’ sense of meaning in life, and is an important way to promote college students’ mental health and enhance their sense of meaning in life. To explore the relationship between stress perception, mental toughness in college students’ physical exercise and college students’ sense of meaning in life. The scale was used to measure 604 college students and statistically analyzed using SPSS29.0 and Process 4.1. The results showed that the direct positive effect of physical exercise on college students’ sense of meaning in life was significant (effect value = 0.00297, accounting for 62.39% of the total effect). Through the independent mediation path, physical exercise can indirectly affect the sense of meaning of life by decreasing stress perception (negative mediation effect value = −0.00040, accounting for −8.40%) and enhancing mental toughness (positive mediation effect value = 0.00127, accounting for 26.68%). In addition, the chain-mediated path (physical exercise → stress perception → mental toughness → sense of meaning in life) effect of stress perception and mental toughness was significant (effect value = 0.00092, accounting for 19.33%), suggesting that physical exercise first relieves stress perception, then enhances mental toughness, and ultimately enhances the sense of meaning in life. To summarize, physical exercise positively predicts the sense of meaning of life through direct action, and also forms a multiple mechanism of “stress reduction - toughness enhancement - meaning enhancement” through the negative intermediary of stress perception, the positive intermediary of mental toughness, and the chain intermediary effect of the two. Meanwhile, it provides theoretical references for the enhancement of college students’ sense of meaning in life and the improvement of sports health promotion programs.

## 1 Introduction

The Global *Physical Activity* Status Report 2022, released by the World Health Organization (WHO), highlights the critical role of physical exercise in promoting individual physical and mental health as well as sustainable social development. College students, as a transitional group preparing to enter society, face a complex array of stressors. These include the intensification of social competition, acceleration of life’s pace, academic burdens, employment pressures, interpersonal challenges, and high self-expectations. According to WHO statistics from 2023, approximately 280 million people worldwide suffer from depression, with over 720,000 suicides annually attributed to it. Prolonged exposure to stress can severely impact college students’ mental health, eroding their enthusiasm for life and diminishing their sense of meaning in life.

The sense of meaning of life, as an important predictor of individual mental health and a deep desire to pursue the value and meaning of one’s life, is crucial for college students. Studies have shown that college students with a high sense of meaning in life are more likely to be optimistic and proactive in self-encouragement, and better adapt to the college life environment (Makola, 2014). On the contrary, the lack of a sense of meaning in life may cause individuals to fall into the pathological state of “passive neurosis” (Frankl, 1985). It is worrying that more than half of college students lack a sense of meaning in life (Li and Lu, 2010), and these students often lack a clear life plan, lose the spiritual motivation and willpower to pursue the meaning of life (Yin, 2016), and are more likely to display psychological problems such as agitation, fear, low self-esteem, and emotional instability, and even have serious tendencies such as suicide, violence, and abuse (Zika and Chamberlain, 2011). In view of this, cultivating a good sense of meaning of life among college students is not only the key to shaping their positive psychological qualities, but also the internal motivation to help them adapt to the role transition smoothly (Xia et al., 2023). The purpose of this paper is to explore the relationship between physical exercise and college students’ sense of meaning in life, and to deeply analyze how physical exercise affects and enhances college students’ sense of meaning in life.

## 2 Theory and assumptions

### 2.1 Effects of physical exercise and sense of meaning in life

Physical exercise, as a kind of physical exercise that integrates a variety of exercise methods, forces of nature and health measures, aims to enhance physical fitness, regulate mental state, and enrich people’s cultural life (Institute HKS, 2000). Sense of meaning in life refers to the extent to which an individual understands and appreciates the meaning of life, and along with it the extent to which he or she perceives the purpose, mission, and primary goal of life (Steger et al., 2006). Research has shown that individuals who regularly engage in physical exercise tend to exhibit more positive psychological states, such as happiness and pleasure, which in turn lead individuals to perceive more meaning in their lives. Park and Folkman (1997) proposed a model of the sense of meaning construct, which describes how individuals create meaning in their lives in different contexts and the relationship between situational meaning and overall meaning. Through automatic and controlled processes, assimilation and regulation, meaning seeking, and cognitive and affective processes, individuals are able to reconstruct their sense of meaning (Park and Folkman, 1997). This model not only provides theoretical guidance for college students in their search for life goals and life values, but also reveals a possible intrinsic link between a sense of meaning in life and physical exercise. Research in exercise psychology further found that college students’ physical exercise is closely related to their sense of meaning in life, and that strengthening physical exercise is conducive to enhancing the level of college students’ sense of meaning in life (Zeng and Zhu, 2021). Based on this, this study proposed Hypothesis H1: Physical exercise positively predicts college students’ sense of meaning in life.

### 2.2 Mediating role of stress perception

Stress perception is the psychological response of an individual to stress after cognitive appraisal, which is manifested as various physical and psychological tensions and discomforts (Li, 2021). According to the cross-stressor adaptation hypothesis of exercise and the stress-buffering role model of exercise (Fuchs and Gerber, 2018; Sothmann et al., 1996), exercise reduces stress perception in several ways. On the one hand, regular participation in physical exercise leads to increased adaptation of the body’s stress response system and reduces an individual’s physiological and psychological responses to other stressful situations, which helps to reduce an individual’s threatening assessment of stressors (Sothmann et al., 1996). On the other hand, physical exercise can enhance stress handling capacity by increasing psychosocial resources such as self-efficacy and social support (Klaperski and Fuchs, 2021). In addition, natural chemicals such as endorphins, which are released by the body during exercise, have a positive effect in relieving stress and anxiety and enhancing mood. Research (Park and Baumeister, 2016) demonstrated that college students’ sense of meaning of life is affected by stress perception, and high stress perception is often accompanied by a low sense of meaning of life. This finding is consistent with previous research findings that psychological stress has a negative effect on an individual’s sense of meaning in life (Schulenberg et al., 2016). Zhou and Zhou (2022) pointed out that appropriate physical exercise can accumulate psychological energy and improve an individual’s ability to resist and adapt to stress. Lu et al. (2009) further emphasized that performing regular physical exercise is more effective in relieving stress, and it can make people feel happy and relaxed, thus effectively releasing stress. Based on this, this study proposes the hypothesis H2: Stress perception plays a mediating role in physical exercise and sense of meaning in life.

### 2.3 The mediating role of mental toughness

Positive psychologist Fred (2008) defines mental toughness as “an individual’s exploitable potential to recover quickly from adversity, failure, positive practices, and increasing responsibilities.” There is a strong correlation between enhancing college students’ sense of meaning in life and having a high level of mental toughness. People need to adapt to some stress or adversity in social environments, which will help individuals to continuously learn to mobilize various resources to cope with stress and subsequently enhance mental toughness (Zhou and Zhou, 2022). Studies on variables related to exercise behavior point out that college students’ goal attitudes have a significant impact on exercise behavior, and emotion plays an important role in forming goal attitudes, which in turn affects the initiation of physical exercise (Nie and Dong, 2015). Physical exercise is regarded as an important influencing factor in related studies on the development of mental toughness. Regular physical exercise can improve mental toughness, and its pathway includes reducing the physical and psychological response to stress, improving the individual’s physiological and psychological state, and acting as a “buffer” for stress (Belcher et al., 2021). A large number of studies have shown that college students’ physical exercise is closely related to the dimensions of goals, cognition, self-efficacy, external support, etc., which have a profound impact on college students’ physical exercise behavior, and mental toughness is highly correlated with these dimensions (Tang and Wang, 2024).

Meanwhile, other studies have shown that mental toughness is positively related to positive life attitudes and the pursuit of life meaning. For college students, clear life goals are a good protective factor for their mental toughness (Feng et al., 2022). College students with a high sense of meaning in life are able to maintain good psychological resilience, which helps them gain a greater sense of control and positive emotions when facing stressful events (Zheng et al., 2023). In turn, the level of psychological resilience can affect the coping styles chosen by individuals in stressful situations, and the higher the level of psychological resilience, the more individuals tend to choose positive coping styles (Liu and Liang, 2019). Based on this, this study proposed hypothesis H3: mental toughness mediates the role of physical exercise and sense of meaning in life.

### 2.4 Chain mediation of stress perception and mental toughness

Research has shown that mental toughness, as a protective mechanism for individuals to cope with stress, can significantly affect people’s responses and adaptive abilities in stressful situations, in which individuals with higher levels of mental toughness are more inclined to adopt effective strategies to reduce or solve stress problems (Zhao, 2020). The cognitive theoretical model of stress reveals that when individuals are in a stressful situation, if they feel out of control and tense, it tends to weaken their ability to mobilize resources to cope with the stress, which reduces the level of mental toughness (Zhang et al., 2017). Stress perception was found to be a significant negative predictor of mental toughness (Liu and Wang, 2016). physical exercise has been shown to be one of the effective ways to reduce stress perception among college students. Regular participation in physical activities not only brings pleasure in exercise and relieves psychological stress caused by academic, social, and other life events, but also makes individuals more comfortable in the face of stress by improving physical function and psychological quality, which in turn reduces the perception of stress and stress response. This process not only directly reduces stress perception, but also indirectly promotes the enhancement of psychological resilience. With the reduction of stress perception, college students are more likely to maintain a positive mindset and increase confidence in facing challenges, and these positive changes provide fertile soil for the growth of mental toughness. Increased mental toughness, as a key mediator connecting physical exercise with a sense of meaning in life, further strengthens the inner strength of college students. Individuals with high mental toughness show greater resilience and growth in the face of life’s ups and downs, and they are more likely to discover the goals and values of life and maintain their passion and pursuit of life. Therefore, through the initial intervention of physical exercise, through the chain reaction of reducing stress perception and enhancing mental toughness, it can eventually significantly enhance college students’ sense of meaning in life and promote their overall healthy development. Based on this, the present study proposed the hypothesis H4: Stress perception and mental toughness may play a chain-mediated role in the effects of physical exercise on college students’ sense of meaning in life.

**Figure 1:**
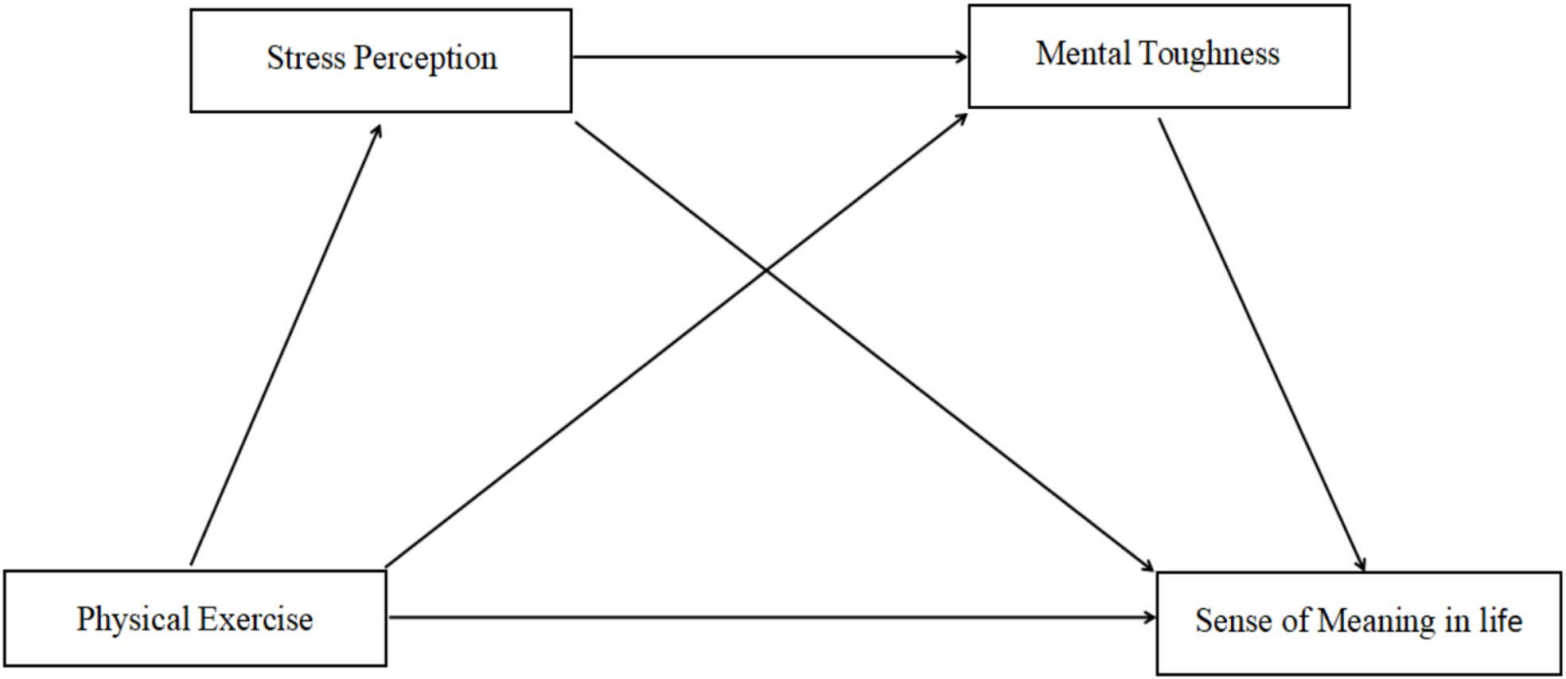
Hypothetical model diagram.

As a result, the research hypothesis model was constructed as shown in Figure 1.

## 3 Research objectives and methods

### 3.1 Participant

In this study, a questionnaire was administered to students from 2 universities in Liaoning Province, China, using stratified random sampling. Subjects were selected from undergraduate level students and consent was obtained from the university and the students themselves prior to testing. The questionnaire followed the principles of voluntary participation, data confidentiality and anonymouscompletion.

After the questionnaires were collected, invalid questionnaires were excluded for data entry, and finally 604 valid data were retained. In the valid sample, four grades of college students were selected as subjects; there were 458 male and 146 female participants. Among the collected questionnaires, questionnaires with any of the following problems were considered invalid: (1) inconsistent responses to reverse scored items; (2) too short completion time; and (3) clear pattern of repeated answer choices.

### 3.2 Research methodology

#### 3.2.1 Physical exercise rating scale

The physical exercise Rating Scale revised by Liang (1994) was used as a tool to assess the physical exercise level of college students. The scale consists of three dimensions, which measure the intensity, frequency and duration of participation in physical exercise, and reflects the individual’s physical exercise level through the comprehensive rating of these three dimensions. For example, the questions, “In the past month, what was the intensity of your physical exercise, in the past month, how often did you engage in physical exercise of the above intensity, and in the past month, how many times did you engage in physical exercise of the above intensity?” were asked on a Likert 5-point scale, with intensity and frequency scored from 1 to 5, respectively. Time was scored from 0 to 4. The amount of physical exercise is equal to the product of physical exercise intensity, physical exercise frequency, and physical exercise time, with higher scores indicating greater physical exercise. The reliability test showed that the Cronbach’s coefficient of the scale was 0.777, indicating that the scale has good internal consistency.

#### 3.2.2 Meaning of life scale

The Sense of Meaning in Life Scale compiled by Steger and revised by Liu and Gan (2010). was used, which contains 2 dimensions of having a sense of meaning and seeking a sense of meaning, with 5 questions for each dimension, for a total of 10 questions. For example, “I am searching for a purpose or mission in my life,” “I have no clear purpose in my life,” and “I am searching for meaning in my life.” The scale is based on a 7-point Likert scale, with higher scores indicating a higher sense of meaning in an individual’s life. The Cronbach’s coefficient for this scale was 0.861, indicating that the scale has good internal consistency.

#### 3.2.3 Stress perception scale

The Stress Perception Scale translated and revised by Yang (2002) was used, which contains 14 questions divided into two dimensions, tension and loss of control. For example, “You feel upset because something unexpected has happened,” “You feel out of control of important things in your life,” and “You feel nervous and stressed.” These questions are scored on a five-point scale from 1 to 5, including seven reverse scored questions, with higher scores indicating greater psychological stress in the subject. The Cronbach’s coefficient for this scale was 0.724, indicating that the scale has good internal consistency.

#### 3.2.4 Mental toughness scale

The Adolescent Mental Toughness Scale developed by Hu and Gan (2008) was used, which contains 27 topics divided into five dimensions: goal focus, emotional control, positive cognition, family support, and interpersonal assistance. For example, “Failure always makes me feel discouraged,” “It is difficult for me to control my unpleasant emotions,” and “I have clear goals in my life,” the scale adopts the 5-point Likert scale scoring The scale was scored on a 5-point Likert scale, with 12 questions reverse scored, with numbers 1 to 5 representing scores in descending order, and the scores were proportional to the subjects’ mental toughness. The Cronbach’s alpha coefficient for this scale was 0.881, indicating that the scale has good internal consistency.

### 3.3 Statistical methods

In this study, Excel was used to enter and organize the data from the questionnaires and screen out invalid questionnaires. SPSS29.0 software, PROCESS plug-in was used to statistically analyze the collected data. Harman’s one-way method was used to test for the presence of common method bias, and Pearson’s correlation analysis was used to explore the relationship between the main variables. In addition, the chain mediation effect was tested with the help of Bootstrap program in PROCESS plug-in.

## 4 Results and analysis

### 4.1 Common method bias test

There may be a problem of common methodological bias in obtaining data from questionnaires due to factors such as the measurement environment, questionnaire guidance and context (Zhou and Long, 2004). The Harman one-way test was conducted on the measurement data, and the unrotated principal component analysis of physical exercise, sense of meaning in life, mental toughness, and stress perception was conducted using SPSS 29.0, and the results showed that there were a total of 10 factors with an eigenroot >1, and the first factor explanatory rate was 23.232%, which was less than the critical value of 40%, indicating that the data of the present study did not suffer from common methodological bias.

### 4.2 Descriptive statistics and correlation analysis for each variable

Descriptive statistical analysis showed that there were 604 university students in the sample, of which 458 (75.8%) were male and 146 (24.2%) were female. This indicates that there are more male students than female students participating in the survey. In terms of grade distribution, the largest number of students were sophomores with 435 students (72.0%), freshmen with 112 students (18.5%), juniors with 41 students (6.8%), and seniors with 16 students (2.6%) (Table 1).

**Table 1:** Distribution of demographic variables among survey respondents.

Causality	Form	Quantities	Percentage
Gender	Male	458	75.8%
	Female	146	24.2%
Grade	1	112	18.5%
	2	435	72.0%
	3	41	6.8%
	4	16	2.6%

An independent samples *t*-test was used to analyze the differences in physical exercise, sense of meaning in life, stress perception, and mental toughness among college students of different genders and grades. The results showed that there was a significant difference in physical exercise at the *α* = 0.05 confidence interval level in the gender subgroups, while there was no significant difference in sense of meaning of life, stress perception and mental toughness in the gender subgroups (Table 2).

**Table 2:** One-sample *t*-tests for physical exercise, sense of meaning in life, stress perception, and mental toughness.

Variant	Gender	*N*	M ± SD	*t*	*p*
Physical exercise	Male	458	43.290 ± 28.961	6.204	<0.001
	Female	146	26.075 ± 29.917		
Sense of the meaning of life	Male	458	3.516 ± 0.791	0.262	0.793
	Female	146	3.497 ± 0.649		
Stress perception	Male	458	2.748 ± 0.485	0.612	0.541
	Female	146	2.720 ± 0.516		
Mental toughness	Male	458	3.470 ± 0.557	−0.468	0.640
	Female	146	3.495 ± 0.591		

**p < 0.01, ***p < 0.001.

ANOVA analysis showed no significant difference in physical exercise and sense of meaning of life on grade level subgroups, and significant difference in stress intuition and mental toughness on grade level subgroups at *α* = 0.05 confidence level as shown in (Tables 3–5).

**Table 3:** ANOVA one-way analysis of variance.

Variant	Grade	*N*	M ± SD	F	*p*
Physical exercise	1	112	42.018 ± 26.644	1.789	0.148
	2	435	37.703 ± 30.610		
	3	41	47.512 ± 31.019		
	4	16	36.188 ± 33.796		
Sense of the meaning of life	1	112	3.604 ± 0.695	1.480	0.219
	2	435	3.509 ± 0.760		
	3	41	3.322 ± 0.898		
	4	16	3.431 ± 0.725		
Stress perception	1	112	2.635 ± 0.577	2.887	0.035
	2	435	2.769 ± 0.471		
	3	41	2.793 ± 0.423		
	4	16	2.594 ± 0.476		
Mental toughness	1	112	3.611 ± 0.589	2.995	0.030
	2	435	3.451 ± 0.555		
	3	41	3.362 ± 0.529		
	4	16	3.479 ± 0.661		

**Table 4:** Multiple comparisons of stress perceptions among college students of different grades.

Grade	1 (42.018)	2 (37.703)	3 (47.512)
4 (36.188)	0.041	0.176	0.199
3 (47.512)	−0.158	−0.023	
2 (37.703)	−0.135		

**Table 5:** Multiple comparisons of mental toughness of college students in different grades.

Grade	1 (42.018)	2 (37.703)	3 (47.512)
4 (36.188)	0.132	−0.028	−0.117
3 (47.512)	0.249	0.089	
2 (37.703)	0.160		

As shown in Table 6, through the Pearson correlation analysis of the data on physical exercise, sense of meaning of life, stress perception and mental toughness, it was found that physical exercise was significantly positively correlated with college students’ sense of meaning of life and mental toughness, physical exercise showed a significant negative correlation with stress perception, college students’ sense of meaning of life was significantly positively correlated with mental toughness, college students’ sense of meaning of life was significantly negatively correlated with stress perception and stress perception has a significant negative correlation with mental toughness. Based on this, hypothesis H1 was verified, which provided support for further construction of structural equation modeling.

**Table 6:** Person correlation analysis.

Variant	Physical exercise	Sense of the meaning of life	Stress perception	Mental toughness
Physical exercise	1			
Sense of the meaning of life	0.180^**^	1		
Stress perception	−0.112^**^	−0.186^**^	1	
Mental toughness	0.189^**^	0.356^**^	−0.708^**^	1

**p < 0.01.

As shown in Table 7, the chain mediation effect was tested using Model 6 in the SPSS PROCESS macro program developed by Hayes. The results showed that:physical exercise had a significant negative predictive effect on stress perception (*β* = −0.126, *p* < 0.01), physical exercise had a significant positive predictive effect on mental toughness (*β* = 0.120, *p* < 0.001), sense of meaning in life (*β* = 0.118, *p* < 0.01), stress perception had a significant negative predictive effect, physical exercise had a significant positive predictive effect on sense of meaning in life (*β* = 0.127, *p* < 0.05), and mental toughness had a significant positive predictive effect on sense of meaning in life (*β* = 0.419, *p* < 0.001). Including stress perception and mental toughness together in the structural equation, physical exercise had a significant positive predictive effect on college students’ sense of meaning in life (*β* = 0.118, *p* < 0.01).

**Table 7:** Regression analysis between variables.

Equation of regression		Overall fit index			Significance of regression coefficient		
Result variable	Variable of prediction	R	R^2^	F	*β*	*t*	*p*
Stress perception	Physical exercise	0.136	0.018	3.767	−0.126	−3.022	0.003^**^
	Gender				−0.058	−1.400	0.162
	Grade				0.055	1.352	0.177
Mental toughness	Physical exercise	0.720	0.518	161.185	0.120	4.070	0.000^***^
	Stress perception				−0.691	−24.130	0.000^***^
	Gender				0.034	1.167	0.244
	Grade				−0.061	−2.158	0.031^*^
Sense of the meaning of life	Physical exercise	0.387	0.150	21.100	0.118	2.970	0.003^**^
	Stress perception				0.127	2.367	0.018^*^
	Mental toughness				0.419	7.714	0.000^***^
	Gender				0.015	0.395	0.693
	Grade				−0.042	−1.108	0.269

*p < 0.05, **p < 0.01, ***p < 0.001.

### 4.3 Mediation effect test

**Figure 2:**
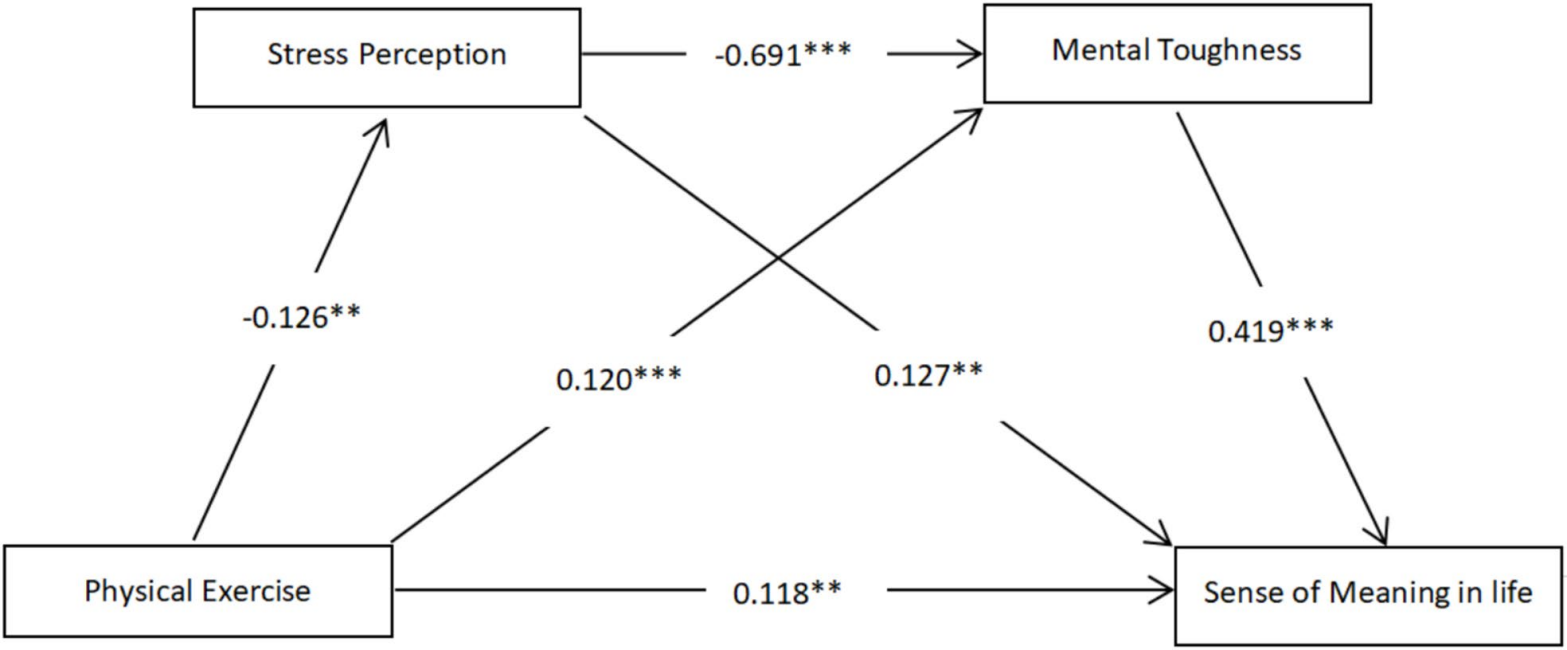
A mediation model of physical exercise that influences college students’ sense of meaning in life (***p < 0.001).

The path coefficients are shown in Figure 2, and the mediating effects of stress perception and mental toughness between physical exercise and sense of meaning in life and the confidence intervals were examined separately by using Bootstrap test with repeated sampling of 5,000 times (Table 8). The results showed that physical exercise produced a total effect value of 0.00476 on college students’ sense of meaning in life, and the 95% confidence intervals of the mediating effects of stress perception and mental toughness did not contain 0, which indicated that the total mediating effect of physical exercise on college students’ sense of meaning in life was significant. The total mediation effect consisted of 3 paths: Ind1: the mediation effect of stress perception in the relationship between physical exercise and college students’ sense of meaning in life, the mediation effect value was −0.00040, the mediation effect accounted for −8.40% of the total effect, and the 95% confidence interval did not contain zero, which indicated that the mediation effect of stress perception was significant. ind2: the mediation effect of mental toughness in the relationship between physical exercise and college students’ sense of meaning in life. Mediating effect, the mediating effect value is 0.00127, the mediating effect is 26.68% of the total effect, and the 95% confidence interval does not contain zero, which indicates that the mediating effect of stress perception is significant. Ind3: Chain mediation of stress perception and mental toughness in the relationship between physical exercise and college students’ sense of meaning in life, the mediating effect value is 0.00092, the mediating effect is 19.33% of the total effect, The 95% confidence interval does not contain zero, which indicates a significant chain mediation effect. The above results indicate that the three indirect effects have reached the significant level and hypothesis 2, hypothesis 3, and hypothesis 4 are valid.

**Table 8:** Proportion of mediating effects.

Effect	Influence path	Effect size	BootSE	BootLLCL	BootULCL	Proportion
Total effect		0.00476	0.00104	0.00271	0.00680	100%
Direct effect	Direct path	0.00297	0.00100	0.00101	0.00494	62.39%
Total indirect effect		0.00179	0.00042	0.00101	0.00265	37.61%
	Ind1	−0.00040	0.00023	−0.00094	−0.00004	−8.40%
Indirect effect	Ind2	0.00127	0.00036	0.00063	0.00201	26.68%
	Ind3	0.00092	0.00035	0.00030	0.00166	19.33%

## 5 Discussion

### 5.1 The relationship between physical exercise and college students’ sense of meaning in life

The results of this study confirm that there is a significant positive correlation between physical exercise and college students’ sense of meaning in life, and physical exercise can positively predict college students’ sense of meaning in life (Liu, 2024). Previous studies have shown (Ding et al., 2016; Takkinen et al., 2001) that strengthening physical exercise inputs can enable college students to experience more enjoyment and fulfillment in life, and then they can perceive the meaning of life more clearly. Physical exercise can not only enhance the physical quality of individuals, but also cultivate college students’ sense of collective honor and responsibility through teamwork and competitive spirit in the process of sports, so that they feel the value and meaning of their own existence, and at the same time, physical exercise also has a positive impact on their emotional health (Anderson and Brice, 2011), so that they can maintain a positive emotional state. And positive emotions are an important foundation for constructing a sense of meaning in life, when college students are in a pleasant mood, they are more likely to think about and explore the meaning of life, and are more likely to connect their behavior with meaningful goals.

### 5.2 The mediating role of stress perception between physical exercise and college students’ sense of meaning in life

The results of this study found that stress perception mediates the relationship between physical exercise and college students’ sense of meaning in life. College life is full of challenges and pressures, including academic burden, employment competition and interpersonal relationships, etc. These pressures, if not released in a timely and effective manner, may lead to psychological problems such as anxiety and depression among college students, which may reduce their sense of meaning in life. When college students are in a high state of stress perception, they tend to feel anxious, confused, and doubtful about their own ability and future, which will greatly weaken their perception of the meaning of life. Excessive stress can cause college students to fall into a negative mode of thinking, focusing only on immediate difficulties and challenges and making it difficult to think about the value and purpose of life from a more macro perspective. However, physical exercise provides an effective way to release pressure. Performing appropriate physical exercise can reduce the emotional experience thereby mitigating the effects of stress, anxiety, and other adverse emotions (Liu C., 2020). Chemicals such as endorphins, which are produced during exercise, help to enhance an individual’s sense of pleasure and wellbeing, thereby reducing stress perception. This stress relief further promotes the positive perception and evaluation of life among college students, who are able to face life with a more positive and rational attitude and have more energy and mental space to explore and pursue the meaning of life. They will be more willing to try new things and participate in a variety of social and learning activities, thus continuously enriching their life experience and thus enhancing their sense of meaning in life.

Mental toughness is a key factor for college students to maintain a positive mindset and optimism in the face of life’s stresses and challenges. Challenges and difficulties in physical exercise can exercise the mental toughness of college students and make them more resilient. This increase in mental toughness helps college students maintain a positive mindset and reduce the impact of negative emotions when facing life’s pressures and challenges. Mental toughness has a positive contribution to college students’ sense of meaning in life. College students with high psychological toughness are able to maintain an optimistic mindset and find positive meaning in difficult situations when facing stress and setbacks in life. They regard setbacks as opportunities for growth, and strive to overcome difficulties and realize their self-worth by constantly adjusting their cognition and behavior. This process of positively coping with setbacks gives them a deeper understanding that the meaning of life lies not only in success and happiness, but also in perseverance and growth in the face of difficulties.

### 5.3 Chain mediation of stress perception and mental toughness between physical exercise and college students’ sense of meaning in life

The results of the chain mediation test showed that stress perception and mental toughness acted as chain mediators between physical exercise and college students’ sense of meaning in life. Physical exercise, as a proven psychological intervention, is important for improving mental health, and sense of meaning of life, as a positive indicator of mental health, is closely related to physical exercise (Wang, 2016). Physical exercise first reduces the perceived level of stress in college students by alleviating their stress perception, which in turn reduces their sense of stress. Subsequently, this reduced sense of stress helps college students maintain a positive mindset in the face of challenges, thereby enhancing their psychological resilience. Finally, the increase in psychological resilience further contributed to college students’ positive appraisal of life and enhanced their sense of meaning in life. This chain-mediated effect reveals the profound impact of physical exercise on college students’ mental health. This result is also similar to a previous study that reported that the presence of a sense of meaning in life was associated with higher levels of positive affect, whereas the search for a sense of meaning in life was not (Barnett et al., 2019). Thus, the presence of a sense of meaning in life is more closely related to an individual’s positive psychological qualities than the search for a sense of meaning in life.

When college students participate in physical exercise, their bodies secrete neurotransmitters such as endorphins and dopamine, which not only regulate emotions, but also help individuals relax, and thus effectively reduce the perception of stress. With the reduction of stress perception, college students gain more security and comfort in their psychology, which creates favorable conditions for the cultivation and development of mental toughness. Mental toughness, as an important psychological quality for individuals to cope with adversity and setbacks, is more likely to be enhanced in this relatively relaxed psychological environment. In the process of physical exercise, college students will face a variety of challenges, such as bottlenecks in the improvement of sports skills, losses in competitions, etc. However, a lower perception of pressure allows them to face these difficulties with a more positive attitude, and through continuous overcoming of difficulties and self-adjustment, mental toughness is gradually enhanced. The enhancement of mental toughness will further have a positive impact on college students’ sense of meaning in life. College students with high mental toughness are able to maintain an optimistic attitude in the face of various uncertainties and difficulties in life, actively seek solutions to problems, and are good at learning lessons from setbacks. This positive coping style enables them to better understand and interpret various experiences in life, thus discovering the meaning and value of life and enhancing the sense of meaning in life.

## 6 Conclusion

Physical exercise significantly enhances college students’ sense of life’s meaning by reducing stress perception and fostering mental toughness. This dual mediation underscores the pivotal role of physical activity in improving psychological resilience and promoting a positive life outlook. Cultivating a sense of meaning in life through physical exercise not only supports mental health but also empowers students to navigate life’s complexities with purpose and resilience.

### 6.1 Limitations

Notably, the study’s cross-sectional design limits its capacity to establish causal relationships, as it captures correlational data at a single time point and cannot definitively determine temporal precedence between physical exercise, stress perception, mental toughness, and sense of meaning in life. Additionally, the sample was exclusively drawn from Liaoning Province, China, which may constrain the generalizability of findings to diverse cultural or regional populations. The reliance on self-report measures also introduces potential limitations, such as social desirability biases or recall inaccuracies, which could affect the objectivity of data collection.

### 6.2 Future research directions

To address these limitations, future studies could adopt longitudinal or experimental designs to track variables over time or manipulate physical exercise interventions, thereby validating causal mechanisms and temporal dynamics. Exploring moderating factors—such as personality traits, academic majors, or socioeconomic status—that may influence the proposed mediating pathways (e.g., how physical exercise impacts stress perception differently among extroverted vs. introverted students) would add nuance to the findings. Such research could help identify contextual conditions under which physical exercise most effectively enhances the sense of meaning in life, contributing to more targeted interventions for diverse college populations. Overall, this study highlights the multifaceted benefits of physical exercise in shaping college students’ psychological wellbeing, while also providing a foundation for future research to deepen our understanding of these relationships and develop culturally adaptive strategies for promoting meaning in life.

## Data Availability

The original contributions presented in the study are included in the article/supplementary material, further inquiries can be directed to the corresponding author.

## References

[ref1] Anderson R. J. Brice S. (2011). The mood-enhancing benefits of exercise: memory biases augment the effect. Psychol. Sport Exerc. 12, 79–82. doi: 10.1016/j.psychsport.2010.08.003

[ref2] Barnett M. D. Moore J. M. Garza C. J. (2019). Meaning in life and self-esteem help hospice nurses withstand prolonged exposure to death. J. Nurs. Manag. 27, 775–780. doi: 10.1111/jonm.12737, PMID: 30481407

[ref3] Belcher B. R. Zink J. Azad A. Campbell C. E. Chakravartti S. P. Herting M. M. (2021). The roles of physical activity, exercise, and fitness in promoting resilience during adolescence: effects on mental well-being and brain development. Biol. Psychiatry 6, 225–237. doi: 10.1016/j.bpsc.2020.08.005, PMID: 33067166 PMC7878276

[ref4] Ding S. Xiao R. Zhang Z. (2016). The relationship between college students' sports activities and sense of meaning in life. Chin. J. Sch. Health 37, 445–448. doi: 10.16835/j.cnki.1000-9817.2016.03.037

[ref5] Feng B. Li Z. Wang K. Cui H. (2022). The relationship between college students' life goals and psychological resilience: the multiple mediating effects of self-control and general self-efficacy. Psychol. Res. 15, 78–85. doi: 10.19988/j.cnki.issn.2095-1159.2022.01.010

[ref6] Frankl V. E. (1985). Man's search for meaning. New York, NY: Simon and Schuster.

[ref7] Fred L. (2008). Psychological capital. Beijing: China Light Industry Press.

[ref8] Fuchs R. Gerber M. (2018). Handbuch Stressregulation und Sport. Cham: Springer.

[ref9] Hu Y. Gan Y. (2008). Development and validity verification of the resilience scale for Chinese adolescent. Acta Psychol. Sin. 40, 902–912. doi: 10.3724/SP.J.1041.2008.00902

[ref10] Institute HKS (2000). Sports science dictionary (elite). Beijing: Higher Education Press.

[ref11] Klaperski S. Fuchs R. (2021). Investigation of the stress-buffering effect of physical exercise and fitness on mental and physical health outcomes in insufficiently active men: a randomized controlled trial. Ment. Health Phys. Act. 21:100408. doi: 10.1016/j.mhpa.2021.100408

[ref12] Li Y. (2021). Application of the Chinese perceived stress scale in representative community adult groups. Chin. Ment. Health J. 35, 67–72.

[ref13] Li X. Lu Q. (2010). A study on the relationship between freshmen's sense of meaning in life and mental health status. Chin. J. Health Psychol. 18, 1232–1235. doi: 10.13342/j.cnki.cjhp.2010.10.037

[ref14] Liang D. (1994). The relationship between college students' stress level and physical exercise. Chin. Ment. Health J. 8, 5–6.

[ref16] Liu C. (2020). The influence of physical exercise on negative emotions in college students: the mediating and moderating effects of self-efficacy and psychological resilience. J. Phys. Educ. 27, 102–108. doi: 10.16237/j.cnki.cn44-1404/g8.2020.05.014

[ref17] Liu Y. (2024). The influence of physical exercise on college students' life meaning sense: chain mediating of personality diathesis and optimistic intelligence quotient. Psychol. Res. 17, 360–366. doi: 10.19988/j.cnki.issn.2095-1159.2024.04.009

[ref18] Liu S. Gan Y. (2010). The reliability and validity of the Chinese version of the meaning of life scale in college students. Chin. Ment. Health J. 24, 478–482.

[ref19] Liu X. Liang R. (2019). A study on the relationship between college students' psychological resilience, self-esteem and coping styles. Educational Theory and Practice 39, 47–49.

[ref20] Liu J. Wang C. (2016). The influence of perceived stress on life satisfaction of vocational college students: the mediating role of resilience. J. Jimei Univ. 17, 49–52.

[ref21] Lu S. Zhang X. Mai Y. Li M. (2009). Study on the impact of physical exercise on psychological stress of college students. J. Capital Univ. Phys. Educ. Sports 21, 352–355.

[ref22] Makola S. (2014). Sense of meaning and study perseverance and completion: a brief report. J. Psychol. Afr. 24, 285–287. doi: 10.1080/14330237.2014.906084

[ref23] Nie H. Dong D. (2015). Research on psychological variables of college students' physical exercise behavior. J. Phys. Educ. 22, 64–68. doi: 10.16237/j.cnki.cn44-1404/g8.2015.05.013

[ref24] Park J. Baumeister R. F. (2016). Meaning in life and adjustment to daily stressors. J. Posit. Psychol. 12, 333–341. doi: 10.1080/17439760.2016.1209542

[ref25] Park C. L. Folkman S. (1997). Meaning in the context of stress and coping. Rev. Gen. Psychol. 1, 115–144. doi: 10.1037/1089-2680.1.2.115

[ref26] Schulenberg S. E. Smith C. V. Drescher C. F. Buchanan E. M. (2016). Assessment of meaning in adolescents receiving clinical services in Mississippi following the Deepwater horizon oil spill: an application of the purpose in life test-short form (PIL-SF). J. Clin. Psychol. 72, 1279–1286. doi: 10.1002/jclp.22240, PMID: 26575078

[ref27] Sothmann M. S. Buckworth J. Claytor R. P. Cox R. H. White-Welkley J. E. Dishman R. K. (1996). Exercise training and the cross-stressor adaptation hypothesis. Exerc. Sport Sci. Rev. 24, 267–287. doi: 10.1249/00003677-199600240-00011, PMID: 8744253

[ref28] Steger M. F. Frazier P. Oishi S. Kaler M. (2006). The meaning in life questionnaire: assessing the presence of and search for meaning in life. J. Couns. Psychol. 53, 80–93. doi: 10.1037/0022-0167.53.1.80

[ref29] Takkinen S. Suutama T. Ruoppila I. (2001). More meaning by exercising? Physical activity as a predictor of a sense of meaning in life and of self-rated health and functioning in old age. J. Aging Phys. Activ. 9, 128–141. doi: 10.1123/japa.9.2.128

[ref30] Tang F. Wang W. (2024). The impact mechanism of college students' psychological resilience on their physical exercise behavior: based on the perspective of habit control and resistance to temptation. J. Phys. Educ. 31, 53–61. doi: 10.16237/j.cnki.cn44-1404/g8.2024.05.008

[ref31] Wang X. (2016). Reliability and validity of the revised Chinese version of the sense of meaning of life scale among college students and its relationship with psychological quality. J. Southwest Univ. 38. doi: 10.13718/j.cnki.xdzk.2016.10.023

[ref32] Xia Q. Liu Q. Song Y. (2023). The relationship between leisure physical exercise and sense of meaning in life among college students: the mediating effects of positive psychological capital and gender differences. J. Hub. Sports Sci. 42, 117–123.

[ref33] Yang T. (2002). Research on the psychological stress of urban population in cities undergoing social transformation. Chin. J. Epidemiol. 23, 64–66.

[ref34] Yin N. (2016). Research on the necessity and implementation approaches of life education for college students. Hangzhou: Zhejiang University of Finance and Economics.

[ref35] Zeng J. Zhu H. (2021). The impact of physical exercise on the sense of meaning in life among college students in Shenzhen. Bull. Sport Sci. Technol. 29, 131–134.

[ref36] Zhang L. Liu W. Guan X. Song W. Zhang Y. (2017). The mediating effect of psychological resilience on nurses' psychological stress and sleep quality. Med. J. Chin. People's Armed Police Forces 28, 261–264. doi: 10.14010/j.cnki.wjyx.2017.03.012

[ref37] Zhao J. (2020). A study on the relationship between college students' psychological resilience, physical activity, and happiness. Tianjin: Tianjin University of Sport.

[ref38] Zheng L. Zheng G. Song X. (2023). Research on the impact of online interpersonal communication on the psychological tesilience of college students: the mediating role of sense of meaning in life and the regulating mechanism of online literacy. J. Chin. Youth Soc. Sci. 42, 61–73. doi: 10.16034/j.cnki.10-1318/c.2023.03.009

[ref39] Zhou H. Long L. (2004). Statistical testing and control methods for common method bias. Adv. Psychol. Sci. 12, 942–950.

[ref40] Zhou H. Zhou Q. (2022). Physical exercise empowers the subjective well-being of college students: the chain mediating effect of cognitive reappraisal and psychological resilience. J. Shandong Sport Univ. 38, 105–111.

[ref41] Zika S. Chamberlain K. (2011). On the relation between meaning in life and psychological well-being. Brit. J. Psychol. 83, 133–145. doi: 10.1111/j.2044-8295.1992.tb02429.x, PMID: 1559144

